# Gradient Poly(ethylene glycol) Diacrylate and Cellulose Nanocrystals Tissue Engineering Composite Scaffolds via Extrusion Bioprinting

**DOI:** 10.3389/fbioe.2019.00280

**Published:** 2019-10-18

**Authors:** Brody A. Frost, Bradley P. Sutliff, Patrick Thayer, Michael J. Bortner, E. Johan Foster

**Affiliations:** ^1^Department of Materials Science and Engineering, Macromolecules Innovation Institute, Blacksburg, VA, United States; ^2^Department of Chemical Engineering, Macromolecules Innovation Institute, Blacksburg, VA, United States; ^3^CELLINK® LLC., Virginia Tech, Blacksburg, VA, United States

**Keywords:** cellulose nanocrystal composites, poly(ethylene glycol) diacrylate composites, pneumatic extrusion bioprinting, gradient scaffolds, bioscaffolds, tissue engineering

## Abstract

Bioprinting has advanced drastically in the last decade, leading to many new biomedical applications for tissue engineering and regenerative medicine. However, there are still a myriad of challenges to overcome, with vast amounts of research going into bioprinter technology, biomaterials, cell sources, vascularization, innervation, maturation, and complex 4D functionalization. Currently, stereolithographic bioprinting is the primary technique for polymer resin bioinks. However, it lacks the ability to print multiple cell types and multiple materials, control directionality of materials, and place fillers, cells, and other biological components in specific locations among the scaffolds. This study sought to create bioinks from a typical polymer resin, poly(ethylene glycol) diacrylate (PEGDA), for use in extrusion bioprinting to fabricate gradient scaffolds for complex tissue engineering applications. Bioinks were created by adding cellulose nanocrystals (CNCs) into the PEGDA resin at ratios from 95/5 to 60/40 w/w PEGDA/CNCs, in order to reach the viscosities needed for extrusion printing. The bioinks were cast, as well as printed into single-material and multiple-material (gradient) scaffolds using a CELLINK BIOX printer, and crosslinked using lithium phenyl-2,4,6-trimethylbenzoylphosphinate as the photoinitiator. Thermal and mechanical characterizations were performed on the bioinks and scaffolds using thermogravimetric analysis, rheology, and dynamic mechanical analysis. The 95/5 w/w composition lacked the required viscosity to print, while the 60/40 w/w composition displayed extreme brittleness after crosslinking, making both CNC compositions non-ideal. Therefore, only the bioink compositions of 90/10, 80/20, and 70/30 w/w were used to produce gradient scaffolds. The gradient scaffolds were printed successfully and embodied unique mechanical properties, utilizing the benefits of each composition to increase mechanical properties of the scaffold as a whole. The bioinks and gradient scaffolds successfully demonstrated tunability of their mechanical properties by varying CNC content within the bioink composition and the compositions used in the gradient scaffolds. Although stereolithographic bioprinting currently dominates the printing of PEGDA resins, extrusion bioprinting will allow for controlled directionality, cell placement, and increased complexity of materials and cell types, improving the reliability and functionality of the scaffolds for tissue engineering applications.

## Introduction

Additive manufacturing (AM) technology, also known as rapid prototyping, was originally introduced toward the end of the 1980s, and has grown substantially in the last few decades (Gebhardt, 2011; Bandyopadhyay and Bose, 2015). The process involves a “bottom-up” approach by adding materials layer by layer to create three-dimensional parts directly from computer-aided design (CAD) models, rather than the typical subtractive manufacturing approach (Gebhardt, 2011; Guo and Leu, 2013; Bandyopadhyay and Bose, 2015). The major AM processes include directed energy deposition, vat polymerization, powder bed fusion, material jetting and extrusion, sheet lamination, and binder jetting. All of these can be sub-sectioned into many other specific categories based on applications for which they are applied (Guo and Leu, 2013; Gibson et al., 2015; Ngo et al., 2018; Tofail et al., 2018). Additionally, AM processes have been expanded to multiple materials ranging from metal alloys, to polymer composites, to ceramics and concrete, lending to the current state of materials development and functionality (Guo and Leu, 2013; Quan et al., 2015; Wang et al., 2017; Ngo et al., 2018). With the many recent improvements in AM technology, a variety of industrial applications are now being discovered and commercialized, including applications in aerospace, automotive, architecture, electronics, medicine/biomedical, and even sports (Guo and Leu, 2013; Tuomi et al., 2014; Ngo et al., 2018; Tofail et al., 2018). Most notably however, is the rise of biomedical applications, such as bioprinting/biofabrication of tissues, orthopedic implants and prosthetics, and regenerative medicine, among many others (Mironov et al., 2006; Murphy and Atala, 2014; Tuomi et al., 2014; Ngo et al., 2018; Tofail et al., 2018). Bioprinting has experienced rapid growth in the last few years, becoming an important aspect in the biomedical field (Mironov et al., 2006; Tasoglu and Demirci, 2013; Murphy and Atala, 2014; Bishop et al., 2017). It utilizes multiple aspects of tissue engineering such as biomimicry, autonomous self-assembly, and mini-tissue building blocks through precise layer by layer positioning of compatible bioinks to produce complex 3D functional living tissues (Tasoglu and Demirci, 2013; Murphy and Atala, 2014; Bishop et al., 2017; Zhang et al., 2018). These bioinks typically consist of biologically compatible materials, with or without seeded cells, in a resin or ink form that can be cast, printed, or otherwise molded, and subsequently crosslinked by a stimulus to create a biomaterial scaffold (Tasoglu and Demirci, 2013; Murphy and Atala, 2014; Bishop et al., 2017; Zhang et al., 2018).

The four main types of bioprinting techniques are laser-assisted bioprinting, inkjet bioprinting, extrusion bioprinting, and stereolithography (SLA), all of which have been heavily studied in the bioprinting field (Guillotin et al., 2010; Iwanaga et al., 2015; Pati et al., 2015; Raman and Bashir, 2015; Bishop et al., 2017; Zhang et al., 2018; Jiang T. et al., 2019). Each of these techniques use unique processes such as thermal, piezoelectric, mechanical, and light energy, to develop complex tissue scaffolds with high resolution (1–500 μm) and high cell viability (80–98%) (Guillotin et al., 2010; Iwanaga et al., 2015; Pati et al., 2015; Raman and Bashir, 2015; Bishop et al., 2017; Zhang et al., 2018; Jiang T. et al., 2019). Although all of these techniques have been used for bioprinting of tissue engineering and regenerative medicine applications, each have their limitations and disadvantages. For example, SLA can only print single photocurable polymer resins with a single cell type, while extrusion bioprinting can print multi-material assemblies with varying cell types (Raman and Bashir, 2015; Bishop et al., 2017; Jiang T. et al., 2019). However, extrusion bioprinting falls short with bioinks needing to possess a certain viscosity in order to hold shape after printing (Pati et al., 2015; Bishop et al., 2017; Zhang et al., 2018; Jiang T. et al., 2019). Therefore, research has been poured into improving these techniques to broaden their capabilities in the bioprinting field (Guillotin et al., 2010; Iwanaga et al., 2015; Pati et al., 2015; Raman and Bashir, 2015; Bishop et al., 2017; Zhang et al., 2018; Jiang T. et al., 2019), with this study focusing on the extrusion bioprinting technique and compatible bioinks.

These printing techniques combined with advancing cell biology and biomaterials have allowed for the progression of tissue engineering and regenerative medicine to applications, such as organ replication and wound repair (Ozbolat and Yu, 2013; Tasoglu and Demirci, 2013; Murphy and Atala, 2014; Irvine and Venkatraman, 2016; Bishop et al., 2017; Zhang et al., 2018). Initially, the challenges facing bioprinting were biological in nature, relating to cell viability and long-term functionality after printing (apoptosis) (Mironov et al., 2006; Tofail et al., 2018; Zhang et al., 2018). Thus, bioprinting began as a way to print complex 3D biocompatible and biodegradable scaffolds that could later be seeded with cells and support matrices such as extracellular matrix (Mironov et al., 2006). Countless research to solve this limitation has since pushed bioprinting techniques past the challenges of integrating cells into bioinks, and toward printing functional tissue scaffolds (Murphy and Atala, 2014; Bishop et al., 2017; Tofail et al., 2018; Zhang et al., 2018). However, these advancements have led to even more challenges, with further progression needed for improved reliability and functionality of bioprinting for major medical applications (Mironov et al., 2006; Murphy and Atala, 2014; Irvine and Venkatraman, 2016; Bishop et al., 2017; Tofail et al., 2018; Zhang et al., 2018). For example, the availability of transplantable organs is drastically lower than the demand for organ transplant patients (Ozbolat and Yu, 2013). The demand of organ transplants in the US in 2015 was 121,070 individuals, while only 2,553 were available, leading to an average of 22 people dying from waiting, per day (Irvine and Venkatraman, 2016). With efficient and effective bioprinting technologies, this major problem could become much less significant in a society with the ability to artificially replicate functional organs (Ozbolat and Yu, 2013; Irvine and Venkatraman, 2016). Thus, research focused on improving bioprinting in specific areas such as bioprinter technology, biomaterials, cell sources, vascularization, innervation, maturation, and complex 4D functionalization is crucial to address the medical problems facing society (Murphy and Atala, 2014).

Recent research has started to explore the areas of embedded stem cells and gradient scaffolds in pursuit of future tissue replacement (Chamberlain et al., 2007; Hwang et al., 2008; Tasoglu and Demirci, 2013; An et al., 2015; Irvine and Venkatraman, 2016; Bracaglia et al., 2017; Moore et al., 2018). The nearly limitless ability of stem cells to differentiate into functional cells promises to contribute to the regeneration of mesenchymal tissues such as bone, cartilage, muscle, ligament, tendon, and adipose, and could lead to much more complex 4D tissue engineering concepts utilizing bioprinting techniques (Chamberlain et al., 2007; Hwang et al., 2008; Tasoglu and Demirci, 2013; Irvine and Venkatraman, 2016; Moore et al., 2018). A few studies by Tasoglu et al., Irvine et al., and Moore et al., have demonstrated the ability and significance of embedding stem cells into 3D bioprinted scaffolds, which could offer great potential for multiple regenerative medicine applications and further development of regenerative therapies (Hwang et al., 2008; Tasoglu and Demirci, 2013; Irvine and Venkatraman, 2016; Moore et al., 2018). With environmental factors playing a major role in stem cell differentiation and growth, gradient (multi-material) scaffolds utilizing different materials and biological components could be of significant importance with regards to complex 4D bioscaffolds for tissue engineering applications (Hwang et al., 2008; Irvine and Venkatraman, 2016; Bracaglia et al., 2017; Moore et al., 2018). Gradient scaffolds have been recently researched for tissue engineering applications in a few studies by Bracaglia et al., An et al., Woodfield et al., and Bittner et al., proving the efficacy of bioprinting complex 4D scaffolds (Woodfield et al., 2005; An et al., 2015; Bracaglia et al., 2017; Bittner et al., 2019). However, these studies, among others, focus mainly on porosity gradients for cell integration and growth, rather than utilizing gradients of materials, stiffness, cell types, and biological factors (Woodfield et al., 2005; An et al., 2015; Bracaglia et al., 2017; Bittner et al., 2019). Furthermore, these studies use SLA bioprinting, or other similar techniques, which only focus on a single cell type and material per scaffold instead of broadening the variety of gradients by other bioprinting techniques such as extrusion bioprinting (Woodfield et al., 2005; Murphy and Atala, 2014; An et al., 2015; Bracaglia et al., 2017; Bittner et al., 2019).

With current trends leading toward gradient scaffold fabrication and stem cell embedment, this paper seeks to further progress the field of bioprinting by fabricating novel poly(ethylene glycol) diacrylate (PEGDA)/cellulose nanocrystal (CNC) bioinks with varying ratios of PEGDA to CNCs, that can be used with a pneumatic extrusion bioprinting method to create single material scaffolds and gradient scaffolds for potential complex 4D tissue engineering applications. PEGDA and CNCs are both biocompatible materials that have been researched extensively for biomedical applications such as tissue engineering and regenerative medicine, however there are still many useful applications that have yet to be explored (Fairbanks et al., 2009; Jaramillo et al., 2012; Dugan et al., 2013; Kumar et al., 2014; Camarero-Espinosa et al., 2016; Palaganas et al., 2017; Jiang Z. et al., 2019; Tang et al., 2019). Most, if not all, of the PEGDA bioscaffolds (Fairbanks et al., 2009; Jaramillo et al., 2012; Palaganas et al., 2017; Jiang Z. et al., 2019; Tang et al., 2019) and CNC-reinforced bioscaffolds (Dugan et al., 2013; Kumar et al., 2014; Camarero-Espinosa et al., 2016; Tang et al., 2019) have been fabricated using SLA and direct light curing due to their low viscosity, only allowing for the use of one material in resin form and one type of cell. Extrusion bioprinting has the ability to overcome these limitations by allowing different print heads to carry different compositions and/or materials and imbedded cell types, increasing the available complexity of the printed scaffolds (Murphy and Atala, 2014). Different ratios of PEGDA to CNCs will be used to tune the viscosity and resulting mechanical properties of each bioink, in order to allow printability of gradient scaffolds varying in composition, stiffness, and hydration. Cast scaffolds will also be fabricated to compare the extrusion printing method with conventional casting and curing methods. These bioinks and printed scaffolds will be rheologically and mechanically tested, respectively, to determine the success of CNC reinforcement within the PEGDA matrix, as well as the variation in properties from a single composition scaffold to a gradient composition scaffold. Although it was not within the scope of this study, embedment of stem cells within each composition of PEGDA/CNC bioinks could prove effective in varying differentiation based on the different stiffness and hydration regions of the gradient scaffolds (Chamberlain et al., 2007; Hwang et al., 2008; Tasoglu and Demirci, 2013; Irvine and Venkatraman, 2016). This ability to incorporate not only stem cells, but also multiple types of cells in varying bioinks using multiple print heads, could be used to increase the availability of tissue engineering applications featuring multi-component tissue replacements, such as ligaments, tendons, and membranes (Hwang et al., 2008; Tasoglu and Demirci, 2013; Murphy and Atala, 2014; Irvine and Venkatraman, 2016).

## Materials and Methods

### Materials

Poly(ethylene glycol) diacrylate (PEGDA, M_n_ 575) and lithium phenyl-2,4,6-trimethylbenzoylphosphinate (LAP) were purchased from Sigma-Aldrich. Commercial sulfated cellulose nanocrystals (CNCs) in the form of an 11.8 wt.% aqueous suspension was purchased from the University of Maine Nanocellulose Facility. The CNCs were extracted from a wood source and characterized with average dimensions of 150–200 nm in length and 5–20 nm in width, sulfur content of 0.94 wt.%, and surface charge density of 330 ± 15 mM/kg cellulose. All printing consumables including UV-resistant amber print cartridges, disposable plastic petri dishes, and 30, 25, and 22 gauge sterile high-precision conical bioprinting nozzles were supplied by CELLINK.

### Fabrication of Bioinks

The 11.8 wt.% aqueous CNC suspension was diluted to a 10 wt.% suspension using DI water for ease of composition calculations (i.e., 11.8 mL of DI water was added to 100 mL of the 11.8 wt.% CNC suspension to create a 10 wt.% CNC suspension). PEGDA was then added to the 10 wt.% CNC suspension to create a 500 mL mixture with ratios of 95/5, 90/10, 80/20, 70/30, and 60/40 w/w PEGDA to CNC mixtures, excluding wt.% of the water. To reduce water content and increase viscosity, the mixtures were dried in an IKA RV 10 Auto Pro V-C Rotary Evaporator set at 20 mbar and 32°C, and rotating at 70 rpm for 1 h. This resulted in gels resembling a thick paste, like that of Elmer's® glue. After rotary evaporation, 0.067 wt.% LAP was added to each gel. The resulting gels of each composition were weighed in the wet state, then air-dried in a fume hood and weighed again to determine the actual composition of the bioinks to be used for printing, shown in Table 1. All bioinks were transferred into laboratory glass containers and stored in the refrigerator until use.

**Table 1:** Final bioink compositions, including wt.% DI water, after 1 h in the rotary evaporator, determined by drying and verified by TGA.

**Composition (w/w PEGDA/CNC)**	**wt.%** **PEGDA**	**wt.%** **CNC**	**wt.%** **Water**
95/5	71.1	3.7	25.2
90/10	46.8	5.2	47.9
80/20	28.6	7.1	64.3
70/30	27.9	11.9	60.1
60/40	20.0	13.0	66.6

**Figure 1:**
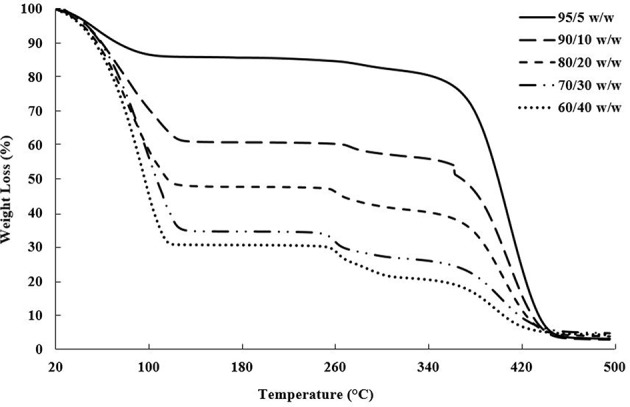
TGA plot of weight loss vs. temperature showing the evaporation of water near 100°C, followed by the CNC degradation between 260 and 280°C and PEGDA degradation between 350 and 450°C (Camarero Espinosa et al., 2013; Reid et al., 2017; Ronca et al., 2018).

The actual bioink compositions, mentioned in Table 1, were verified using thermogravimetric analysis (TGA). Ten milligram of each bioink was measured into a platinum TGA pan, and heated from 25 to 500°C at a rate of 10°C/min using a TA Instruments TGA Q500 thermal analyzer. The bioink compositions were determined based on the weight left after complete water loss, shown in Figure 1.

### Rheology

Rheology measurements were performed on an TA Instruments ARG2 torsional rheometer with a 40 mm plate upper geometry and a Peltier plate lower geometry with a trim gap of 1,050 μm and a testing gap of 1,000 μm. All measurements were carried out at 25°C with a solvent trap. Each sample was pre-sheared at 1 s^−1^ for 30 s and allowed to equilibrate for 5 min. Yield stress measurements were conducted with a stress sweep, applied from 0.01 to 1,000.0 Pa with 10 points per decade at a frequency of 1.0 Hz with 10 samples taken per cycle. Steady shear measurements were conducted over the range 0.005–100.0 s^−1^ with 10 points per decade with a tolerance of 5% torque for three consecutive cycles. A 5 s delay was implemented to avoid rate ramp artifacts, and measured results were averaged over a 30 s time period. Yield stress values were calculated using the Trios onset function. A power-law model (Equation 1) was fit to each set of shear sweep data, where the power-law is traditionally used to model the shear-thinning region of polymeric fluids. In this model η is the viscosity, K is the flow consistency index, $\dot{\gamma }$ is the shear rate, and *n* is the flow behavior index.

(1) $$\begin{array}{l} \eta =K{\dot{\gamma }}^{n-1} \end{array}$$

### Casting PEGDA/CNC Scaffolds

Two methods were used for preparing the cast PEGDA/CNC scaffolds: casting and curing in a Teflon petri dish, and injecting and curing in between glass microscope slides.

The casting process was made with the intention of determining the curing ability and swelling/drying capabilities of the bioink scaffolds. A 5 mL syringe, without the needle, was used to extract 5 mL of each PEGDA/CNC bioink composition. Each bioink was then extruded into a 60 mm Teflon petri dish and let to sit for 1 h. This allowed ample time for the bioinks to settle and ensure that the surfaces were flat and uniform. It should be noted that the bioinks remained stable over the hour and no settlement of CNCs was observed. UV initiating light from a UVP UVL-21 compact UV lamp with a wavelength of 365 nm at 4 W, 0.16 A, 115 V, and 60 Hz was applied directly to each bioink for 60 s to ensure full curing, following an adapted procedure from Fairbanks et al. (2009).

The injection process was made with the intention of making usable cast bioink scaffolds for mechanical testing. Two 76 × 51 mm plain glass microscope slides were separated using 1.0 mm thick Teflon sheeting on each end, while the center region remained open. Each end was then clamped with a RV 05.10 balljoint clamp and tightened to ensure no slippage would occur and uniformity throughout the scaffold. As above, a 5 mL syringe, without the needle, was used to extract 5 mL of each PEGDA/CNC bionink composition. Each bioink was then injected in the open center between the two glass slides. Although 5 mL of the bioink was not needed to fill in between the glass slides, all of the material was injected to ensure removal of all air bubbles and potential defects. Once a uniform film was obtained, the bioink was cured using the previously mentioned curing technique, following an adapted procedure from Fairbanks et al. (2009). The samples were then removed from the glass slides, and cut into 5.0 mm wide ribbons for future mechanical testing.

Swelling and drying tests were performed on the cast scaffolds to determine shrinking and permanent deformation with environmental changes. Each bioink scaffold that was cast and cured in the Teflon petri dishes was removed and immediately imaged. They were then left to dry for 24 h in a fume hood, and subsequently imaged again. After the drying process, each scaffold was submerged into 100 mL of DI water and left for another 24 h before being imaged again.

### Bioprinting PEGDA/CNC Scaffolds

Each bioink composition was loaded into a 3 mL UV-resistant amber print cartridge (CELLINK, Blacksburg, VA), and capped on both the open end and nozzle end to prevent the bioinks from leaking and losing water. All scaffold printing used a three-printhead printer (CELLINK BIO X, Blacksburg, VA).

Preliminary printing using a generic rectangle configuration with a rectilinear infill pattern of 25% was conducted to determine the highest resolution printing parameters. Each composition started under the same initial printing conditions of 25°C under air, 20 kPa of pressure, and extrusion rate of 10 mm/s using a 30-gauge nozzle. If the bioink was not successful in extruding through the 30-gauge nozzle using the initial conditions, the pressure was increased in increments of 5 kPa until a maximum of 75 kPa was reached. If there was still no success of extrusion at 75 kPa of pressure, the nozzles were changed to a higher gauge (25-gauge, then 22-gauge). The pressure settings were returned to initial conditions and the 5 kPa incremental increase was repeated until the extrusion was successful. The final high resolution printing parameters determined the ideal nozzle gauge to be 25-gauge, with pressure continually decreasing as CNC content increased from compositions of 90/10–60/40 w/w, respectively, and were used as the initial printing conditions for the bioink scaffolds.

#### Single Material Scaffolds

A basic rectangle scaffold of dimensions 35 × 7 × 1 mm was designed using Autodesk Fusion 360 AutoCAD software, with the intention of being used for tension testing during mechanical characterization. Each single material scaffold was printed using a single composition of bioink, except for the 95/5 w/w, which showed poor printing properties. The printing conditions were initially set at the highest resolution parameters, and were adjusted based on how the material extruded during printing. As previously determined, a 25-gauge nozzle was used for all bioink compositions, and pressures were set at 45, 20, 15, and 12 kPa, decreasing as CNC content increased from compositions of 90/10–60/40 w/w, respectively. It should be noted that the printing resolution increased as well as, with increasing CNC content, further discussed in the rheology data, section Rheological Properties of Bioinks. Each scaffold composition was printed at least three times using a grid infill pattern of 100% at 25°C under air, and UV-cured using a Gesswein 110V, 365 nm UV post-curing chamber for 60 s following an adapted procedure from Fairbanks et al. (2009). Each scaffold was stored in a container filled with 50 mL of DI water until time of mechanical testing.

#### Gradient Scaffolds

As with the single material scaffolds, a basic rectangle scaffold of dimensions 35 × 7 × 1 mm was used for each gradient scaffold in order to maintain uniformity during mechanical characterization. However, the rectangular scaffolds were split into different designs, with each section of the design containing a different bioink composition, shown in Table 2. The four different designs include a three-layered rectangle, a two-layered rectangle, a three-sectioned rectangle, and a two-sectioned rectangle, shown in **Figures 5**, **8**. The compositions of 90/10, 80/20, and 70/30 w/w were the three chosen based on the mechanical properties determined from tension testing of the single material scaffolds. The 95/5 w/w was unable to print and the 60/40 w/w was too brittle to test reliably.

**Table 2:** The compositions of different sections used for each gradient scaffold.

**Scaffold Design**	**Layer/Section 1** **(w/w PEGDA/CNC)**	**Layer/Section 2** **(w/w PEGDA/CNC)**	**Layer/Section 3** **(w/w PEGDA/CNC)**
Three-layers	90/10	80/20	70/30
Two-layers	90/10	80/20	N/A
Three-sections	90/10	80/20	90/10
Two-sections	90/10	80/20	N/A

It should be noted that each of the three available print heads contained one composition of bioink as follows: print head 1 = 90/10 w/w; print head 2 = 80/20 w/w; and print head 3 = 70/30 w/w. Additionally, the BIO X printer software was not yet capable of printing with multiple print heads from an AutoCAD assembly design STL file. Therefore, since multiple print heads were used for printing the gradient scaffolds, the AutoCAD design needed to first be exported to Slic3r, and assembled into a workable multi-material scaffold capable of using three print heads. After the scaffold was reworked in Slic3r, the design was exported as a GCode file and transferred to the BIO X printer. At least three of each gradient scaffold was printed using the same parameters as the single material scaffolds, with a grid infill pattern of 100% at 25°C under air. Each scaffold was then UV-cured using the previously mentioned curing technique for the single material scaffold, following an adapted procedure from Fairbanks et al. (2009). The scaffolds were subsequently stored in a container filled with 50 mL of DI water until time of mechanical testing.

### Mechanical Testing and Characterization

After the printed samples had been cured, three of each composition from both single material scaffolds and gradient scaffolds, as well as three of each cast scaffolds were mechanically tested utilizing a TA Q800 Dynamic Mechanical Analyzer (DMA). Each sample was tested in tension using an isostatic force test with a force ramp rate of 3 N/min at 25°C until either the scaffold fractured or a maximum of 18 N was reached. It should be noted that all samples remained submerged in DI water to stay hydrated until time of testing.

## Results and Discussion

### Bioink Compositions

PEGDA and CNCs were chosen as the polymer matrix and reinforcing agent, respectively, for the composite bioscaffolds due to biocompatibility, tunability, and extensive research on the materials (Fairbanks et al., 2009; Jaramillo et al., 2012; Dugan et al., 2013; Kumar et al., 2014; Camarero-Espinosa et al., 2016; Palaganas et al., 2017; Jiang Z. et al., 2019; Tang et al., 2019). PEGDA shows very low viscosity at room temperature, limiting this material to SLA bioprinting and other similar techniques. Almost all related studies have shown success with printing PEGDA scaffolds for biomedical applications using SLA, however this technique only allows for one material resin and one cell type to be printed per scaffold (Fairbanks et al., 2009; Jaramillo et al., 2012; Raman and Bashir, 2015; Palaganas et al., 2017; Jiang T. et al., 2019; Jiang Z. et al., 2019). CNCs have shown the ability to vary mechanical properties of polymer composites and scaffolds based on varying content within the polymer matrix (Jorfi et al., 2013; Camarero-Espinosa et al., 2016; Sapkota et al., 2016; Smyth et al., 2017; Frost and Foster, 2019; Tang et al., 2019). The addition of CNCs will therefore help to increase viscosity and stiffen the bioinks in order to limit the deformation and help to maintain their structure before photocrosslinking (Zhou et al., 2011; Ben Azouz et al., 2012). This ability to hold their shape is crucial to expanding the printability of PEGDA to extrusion bioprinting techniques, which will lead to more complex printing structures compared to SLA, such as gradient scaffolds using multiple materials and cell types. As well, the ability to use multiple printheads of an extrusion printer, each containing bioinks of varying CNC content, allows for the fabrication of gradient scaffolds with varying stiffness, and potential incorporation of multiple types of cells that can be specifically placed among the scaffold (Bittner et al., 2019). In further comparison, gradient scaffolds with regards to porosity can be obtained through SLA, while scaffolds with stiffness gradients and multiple cell types cannot (Woodfield et al., 2005; An et al., 2015; Bracaglia et al., 2017; Bittner et al., 2019). Therefore, these bioink compositions could progress the field of extrusion bioprinting.

### Bioink Characterization

#### Composition of Bioinks

Drying tests and TGA were performed to determine the water content, and subsequent dry weight of PEGDA and CNC within the bioinks, shown in Table 1; Figure 1. A few trends were observed with varying CNC concentration, including higher water content and char yield with increasing CNC content, while maintaining nearly identical degradation onsets of each component in the bioink. Polymer composites using CNCs as a reinforcing agent have been shown in literature to increase water absorption with increasing CNC content, even for hydrophobic polymer composite systems (Mendez et al., 2011; Smyth et al., 2018; Frost and Foster, 2019). As water comes into contact with the CNC network, the hydrogen bonds between the CNCs are broken leading to increased space and swelling within the composite or bioink (Mendez et al., 2011; Sapkota et al., 2016; Smyth et al., 2018; Frost and Foster, 2019). With regards to the onset of thermal degradation, the expected results agreed with the observed trends, in which each bioink component degraded within its respective range [i.e., water near 100°C, sulfated CNCs between 260 and 280°C (Camarero Espinosa et al., 2013; Reid et al., 2017), and PEGDA between 350 and 450°C (Kurdikar and Peppas, 1995; Ronca et al., 2018)]. It should be noted that at the 90/10 w/w composition, the bioink goes through a transitioning point from low to high viscosity and develops agglomerates of separate discrete phases within the PEGDA, discussed further in section Rheological Properties of Bioinks. It is assumed that the clumps lead to the discontinuity in weight loss during the onset of PEGDA degradation.

#### Rheological Properties of Bioinks

**Figure 2:**
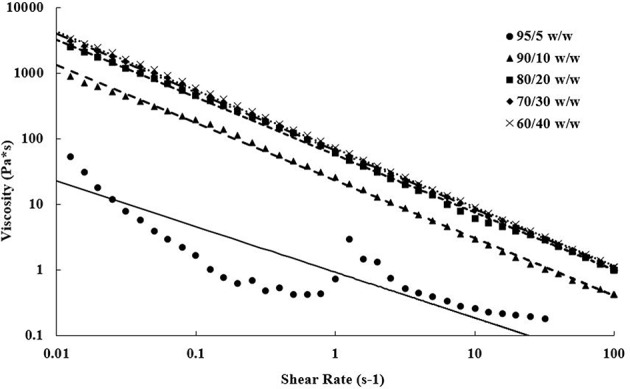
Steady shear viscosity as a function of shear rate showing shear thinning properties of each bioink over ~4 decades. Increasing viscosities are observed as CNC content increases in the bioink compositions. Data points represent actual data, and lines are power-law model fits.

Steady shear torsional rheometry of the bioinks revealed shear thinning behavior of the PEGDA/CNC suspensions over all measured rates. This behavior is typical of CNCs within a suspension or viscous matrix, such as a melt or a gel, in which shear thinning increases as shear rate increases due to the alignment of the CNCs in the shear direction (Shafiei-Sabet et al., 2012; Khabibullin et al., 2017; Fallon et al., 2019). Figure 2 displays the viscosity as a function of the shear rate for each of the bioinks. As expected, higher CNC loading corresponds to higher viscosities, however, above 20 wt.% CNC there is minimal change in steady shear behavior. The 95/5 w/w PEGDA/CNC sample presented issues due to its low viscosity, approaching the lower load limit of the transducer. Figure 2 also displays the power-law model (Equation 1) fits for each composition. The flow consistency index and flow behavior index are provided in Table 3. Aside from the 95/5 composition, the flow index was similar at ~0.11 for all samples, suggesting similar shear thinning behavior across all compositions. Furthermore, the flow consistency index reflects the increasing zero-shear viscosity of the samples as CNC content is increased. Table 3 also presents the yield stress for each sample, which increase with CNC content. A higher yield stress should withstand higher load before induced flow, allowing for more layers to be deposited before photo-curing is necessary to retain the scaffold shape. While this is beneficial for printing taller scaffolds quickly, it also necessitates higher stresses on any cells that may be printed at the same time. As a result, tuning of these parameters is necessary for successful printing of both the geometry and the cell lines. Similarly, both the flow rate and the corresponding viscosity of the bioink must be carefully tuned to control road volume and to avoid pressure buildup within the printer.

**Table 3:** Yield stress and power law model parameters for each bioink composition.

**Composition (w/w PEGDA/CNC)**	**Yield Stress (Pa)**	**Flow Consistency Index, K (Pa·s^**n**^)**	**Flow Behavior Index, *n***
95/5	0.79	0.95	0.37
90/10	37.75	23.08	0.12
80/20	40.09	56.41	0.12
70/30	57.63	65.57	0.11
60/40	60.94	70.67	0.10

### Cast PEGDA/CNC Scaffolds

**Figure 3:**
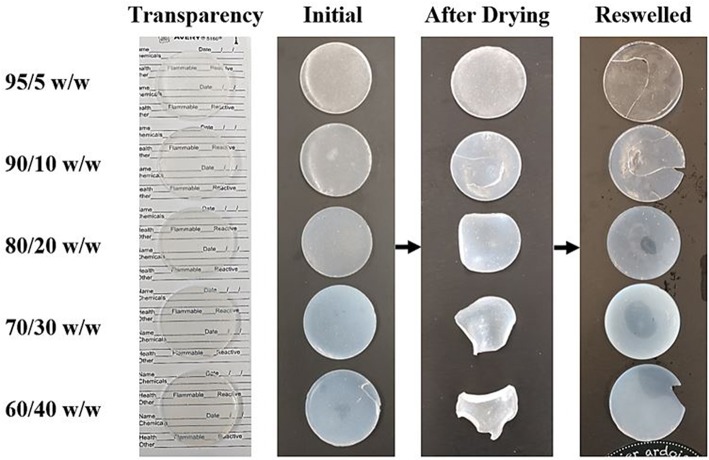
An image showing the optical properties and drying and swelling properties of each composition of cast scaffolds. The arrows show the scaffolds going from initially crosslinked to dried back to swelled. Deformation of the dried scaffold increases as CNC content increases. Opacity also increases with CNC content, however, the scaffolds still remain translucent in the swelled state (left most image).

Each composition of bioink was cast into a Teflon petri dish and cured using UV light, resulting in the disc-shaped scaffolds shown in Figure 3. Drying and swelling tests revealed that the higher CNC content scaffolds (80/20, 70/30, and 60/40 w/w) start to deform and actuate when dried, and fully recover their initial shape after subsequent swelling. The lower CNC content scaffolds (95/5 and 90/10 w/w) showed less deformation and change during drying and swelling, maintaining their disc-like shape throughout the process, however, fracture occurred during swelling from rapid expansion of the brittle cross-linked PEGDA (Khandaker et al., 2016; Zhu et al., 2017). It is believed that as CNC content increases, the crosslinking of the PEGDA is increasingly inhibited, leading to shorter chains and networks throughout the composite. This phenomenon induces greater strains on the longer, less mobile networks of chains when the lower CNC compositions are swelled (Castro et al., 2013). This leads to a higher chance of fracture, unlike more elastic polymers such as polyurethane, where the swelling of the composite would result in no fractures (Frost and Foster, 2019). As well, the drying and reswelling properties themselves showed novelty compared to typical CNC hydrogels found in literature (Yin et al., 2014; Li et al., 2018; Jayaramudu et al., 2019). Usual trends observe that as CNC content within the scaffolds increases, specifically above 5 wt.%, the swelling properties drastically decrease and lead to further embrittlement (Yin et al., 2014; Li et al., 2018; Jayaramudu et al., 2019). The composite scaffolds in this study showed a surprisingly opposite effect, in which additional CNC content resulted in higher swelling properties and lower fracture potential upon drying and reswelling. These unique properties can be attributed to efficient dispersion of CNCs within the scaffolds and increased defects in the PEGDA crosslinking due to higher inhibition from increased CNC content, leading to additional free volume within the scaffold. The optical properties also fluctuated with varying CNC content, increasing in opaqueness as CNC content increased (Frost and Foster, 2019). Although color and transparency varied between the scaffold compositions, they all demonstrated enough transparency to see lettering underneath, shown in Figure 3.

### Bioprinted PEGDA/CNC Scaffolds

Scaffolds of each bioink composition and gradients of bioink compositions were 3D bioprinted using a three-headed printer (CELLINK BIO X, Blacksburg, VA). Unlike the casting technique, the bioprinting technique allowed for ordered scaffolds with specific infill patterns and CNC alignment with subsequent crosslinking alignment of PEGDA. When the bioink is extruded through the print head nozzles, they experience a shear stress that aligns the CNCs in the direction of the print head movements (Walther et al., 2011; Siqueira et al., 2017). The alignment of CNCs can be utilized to design complex hierarchical structures leading to directionality of mechanical properties and, when introduced, specific cell alignment (Walther et al., 2011; Bourget et al., 2013; Siqueira et al., 2017). This alignment also creates a larger barrier in one direction due to the aspect ratio of the CNCs, inhibiting the crosslinking of PEGDA in certain directions, leading to a general alignment of the crosslinked networks in the scaffolds (Lin et al., 2005; Kashima et al., 2010). This can be compared to the typical SLA technique, which exhibits random CNC orientation within the scaffold from the dispersion in the liquid polymer resin (Raman and Bashir, 2015; Palaganas et al., 2017; Jiang T. et al., 2019). These comparisons can also be applied to cells and other biological components within the scaffolds, in which extrusion bioprinting has the ability to align and place in specific locations (Bourget et al., 2013; Murphy and Atala, 2014; Jiang T. et al., 2019; Tang et al., 2019).

**Figure 4:**
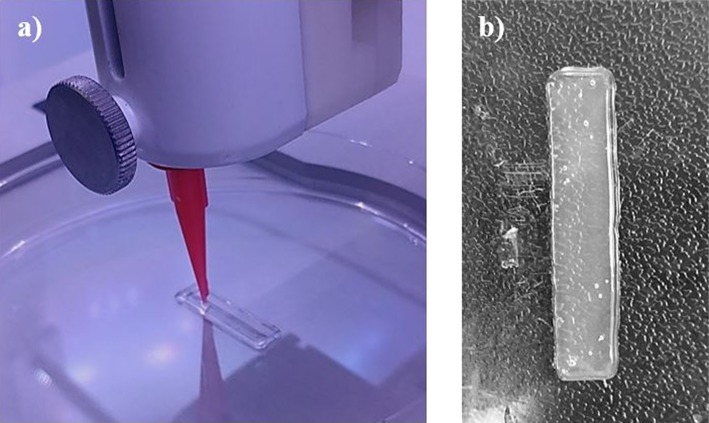
Images depicting **(a)** the printing process of each scaffold, laying down the perimeter and grid infill pattern, and **(b)** an example of the single material scaffolds (90/10 w/w) after crosslinking via UV light. Smooth and uniform dimensions were produced from printing single material scaffolds.

**Figure 5:**
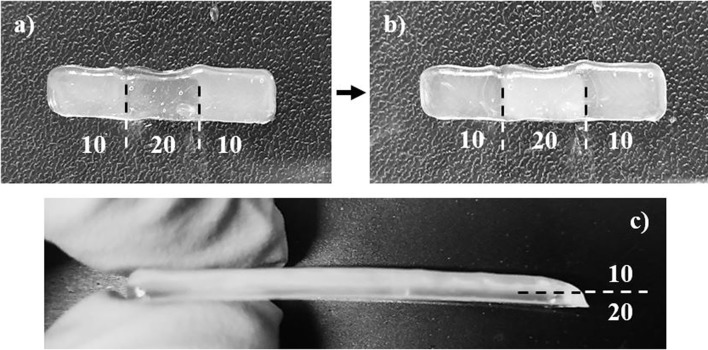
Images depicting **(a)** the 10/20/10 (by wt.% CNC) sectioned scaffold before and **(b)** after crosslinking via UV light, and **(c)** the 10/20 (by wt.% CNC) layered scaffold after crosslinking via UV light. The sectioned scaffold was less uniform due to small calibration errors in the print heads, and demonstrates unique optical properties, switching translucency of compositions after crosslinking. The layered scaffold stayed relatively uniform, and the multiple layers can be seen.

Each single material scaffold was printed using the parameters listed in section Single Material Scaffolds, however the 95/5 w/w bioink was unable to print due to the low viscosity. Instead of holding its shape after printing, the low surface energy caused the bioink to adhere and wet the surface. The single material scaffolds showed a higher uniformity in its dimensions compared to both the cast and gradient scaffolds, shown in Figures 4, 5a. Since the cast scaffolds were cut into strips using a razor blade, the brittleness of the crosslinked PEGDA led to rough, defected edges, while the printer was able to lay down a much smoother perimeter of bioink to hold the dimensions of the scaffolds before crosslinking.

The gradient scaffolds were printed using the parameters listed in section Gradient Scaffolds. Although the gradient scaffolds were printed using the same parameters as the single material scaffolds, they showed a decreased uniformity due to the use of multiple print heads, shown in Figure 5a. Each print head had to be calibrated identically. However, the calibration was manually performed, therefore the dimensions were not as uniform as the single material scaffolds. The layered gradient scaffolds showed higher uniformity, similar to that of the single material scaffolds, shown in Figure 5b. Although the dimensions were slightly less uniform, the gradient scaffolds showed distinct regions in which different bioink compositions were used. Unfortunately, even with distinct regions printed, the relatively low yield stress properties of the bioinks caused the different compositions to flow slightly into one another. This creating crosslinking between the sections and layers. Additionally, the 90/10 and 80/20 w/w compositions, shown in Figure 5a, demonstrated a unique optical property, switching translucency after crosslinking (Liu et al., 2010).

### Mechanical Testing and Characterization

Three samples of each composition of cast, single material, and gradient material scaffolds were mechanically tested and characterized using a controlled force ramp in a DMA, resulting in the mechanical properties shown in Table 4. Throughout all of the samples, a general trend was observed in which the elastic moduli and yield stresses decreased as CNC content increased. The strain at break however, showed no observable trend for the cast scaffolds, and a decreasing trend with additional CNC content for the single material scaffolds. The gradient scaffolds showed unique characteristics and trends, borrowing certain properties from each composition of bioinks used, which is typical for multi-material composites (Jones, 1999; Gay, 2015; Camarero-Espinosa et al., 2016; Gao et al., 2016).

**Table 4:** Mechanical properties of the cast, single material, and gradient scaffolds as determined by DMA.

**Composition**	**95/5 w/w**	**90/10 w/w**	**80/20 w/w**	**70/30 w/w**	**60/40 w/w**
**Cast Scaffolds**					
Elastic modulus (MPa)	27.8 ± 5.8	18.3 ± 2.5	9.6 ± 0.5	1.7 ± 0.1	0.4 ± 0.1
Yield stress (MPa)	0.7 ± 0.1	0.5 ± 0.1	0.4 ± 0.1	0.2 ± 0.1	0.1 ± 0.0
Strain at break (%)	12.9 ± 11.3	14.2 ± 9.6	7.5 ± 3.5	14.5 ± 6.3	22.9 ± 8.9
**Single Material Scaffolds**					
Elastic modulus (MPa)	N/A	16.5 ± 3.0	7.5 ± 3.3	2.7 ± 0.2	0.9 ± 0.1
Yield stress (MPa)		0.3 ± 0.1	0.1 ± 0.1	0.1 ± 0.0	0.1 ± 0.0
Strain at break (%)		46.7 ± 22.3	22.1 ± 4.8	15.5 ± 8.9	8.1 ± 2.3
Composition^*^		**10/20/30** **layered**	**10/20** **layered**	**10/20/10** **sectioned**	**10/20** **sectioned**
**Gradient Material Scaffolds**					
Elastic modulus (MPa)		11.3 ± 0.7	16.7 ± 1.3	14.7 ± 2.4	12.1 ± 2.6
Yield stress (MPa)		0.3 ± 0.1	0.1 ± 0.0	0.2 ± 0.0	0.1 ± 0.0
Strain at break (%)		40.9 ± 30.2	8.7 ± 3.4	17.6 ± 8.5	8.0 ± 3.6

* *As previously discussed in section Gradient Scaffolds, the numbers refer to the wt.% CNC in the composition*.

#### Cast Scaffolds

**Figure 6:**
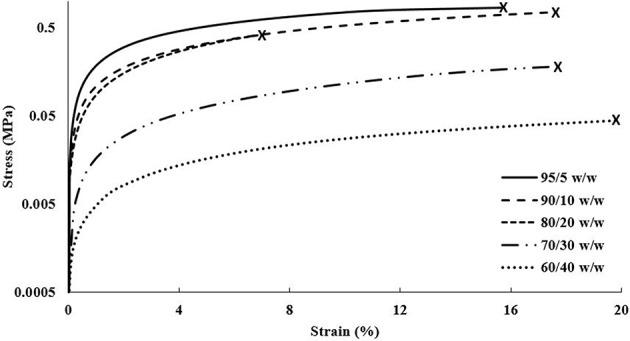
DMA stress vs. strain plot of each composition of cast scaffolds showing strain until break.

The DMA data acquired for the cast scaffolds during tensile testing revealed a general trend of decreasing elastic moduli and tensile yield stresses with increasing CNC content, shown in Figure 6. It should be noted that the elastic moduli were determined by the taking the slope of the linear viscoelastic region and tensile yield stresses were determined by the break in linearity from the linear viscoelastic region. Although this disagrees with most literature, in which CNCs typically increase the mechanical integrity of the composites (Jorfi et al., 2013; Xu et al., 2013; Kargarzadeh et al., 2015; Camarero-Espinosa et al., 2016; Sapkota et al., 2016; Smyth et al., 2017; Frost and Foster, 2019), these observations can be explained by the unique crosslinking inhibition of the CNCs in PEGDA (Lin et al., 2005; Kashima et al., 2010). As the CNC content increases, the PEGDA chains have less ability to form crosslinked networks from the barrier formed by the CNCs (Lin et al., 2005; Kashima et al., 2010; Khan et al., 2012). Additionally, the cast scaffolds maintained random orientation of CNCs within the PEGDA, causing the lower CNC compositions (95/5, 90/10, and 80/20 w/w) to stay under the percolation threshold (Frost and Foster, 2019). This allowed the cast scaffolds to be slightly more reinforced than their single material scaffold counterparts, discussed in section Single Material Scaffolds. However, as the CNC content increased into the 30 wt.% regime, the random orientation of the CNCs caused the scaffolds to pass the percolation threshold isotropically, resulting in defects and lower mechanical properties (Frost and Foster, 2019). The strain at break showed no specific trend with increasing CNC content, however the 80/20 w/w scaffolds showed the lowest value of 7.5 ± 3.5%, while the other compositions were at minimum above 10%.

#### Single Material Scaffolds

**Figure 7:**
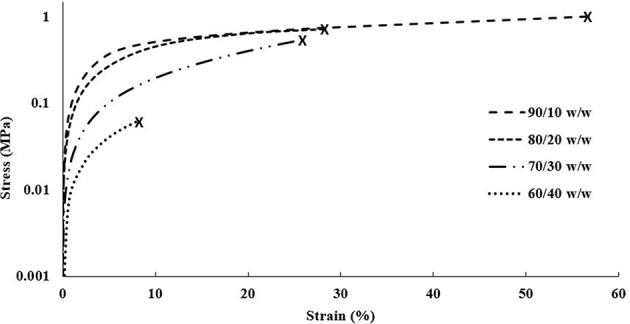
DMA stress vs. strain plot of each composition of single material scaffolds showing strain until break.

The DMA data obtained for the single material scaffolds revealed a similar trend to the cast scaffolds, decreasing in elastic moduli and tensile yield stresses with increasing CNC content, shown in Figure 7. Like the cast scaffolds, this trend disagrees with most literature reporting increased tensile mechanical reinforcement with increasing CNC content (Jorfi et al., 2013; Xu et al., 2013; Kargarzadeh et al., 2015; Camarero-Espinosa et al., 2016; Sapkota et al., 2016; Smyth et al., 2017; Frost and Foster, 2019). As previously discussed, this is believed to be caused by the increase of CNCs inhibiting the crosslinking ability of the PEGDA (Lin et al., 2005; Kashima et al., 2010). However, another general trend was observed that agrees with most literature, in which the strain at break decreased with increasing CNC content (Jorfi et al., 2013; Xu et al., 2013; Kargarzadeh et al., 2015; Sapkota et al., 2016; Frost and Foster, 2019). The 90/10 w/w showed the best mechanical properties of higher reinforcement and strain at break compared to all other compositions. Although the elastic moduli for the 90/10 and 80/20 w/w compositions were slightly below the cast scaffolds, the higher CNC content of 70/30 and 60/40 w/w compositions showed almost double the elastic moduli in comparison. Further, the 90/10, 80/20, and 70/30 w/w compositions showed higher strains at break than their cast scaffold counterparts. The tensile yield stresses of all compositions, however, maintained lower values than the cast scaffolds. These changes in mechanical properties may be caused by the alignment of the CNCs within the printed scaffolds (Walther et al., 2011; Siqueira et al., 2017). As previously discussed, when the bioink is extruded through the print head, the CNCs undergo shear stresses that align them in the direction of the nozzle and its motion (Walther et al., 2011; Siqueira et al., 2017). Since a grid infill pattern was used for the single material scaffolds, printing at ± 45° angles to the perimeter, the mechanical properties followed the trends of a traditional composite material at those same angles, such as carbon fiber composites made with alternating sheet angles (Nak-Ho and Suh, 1979; Pereira and de Morais, 2004; Lee et al., 2007). This allowed for nearly identical mechanical reinforcement properties as the cast scaffolds, however, drastically improved the strain at break for the lower CNC compositions, due to the “scissor” effect (Snell, 1978; Pereira and de Morais, 2004; Botelho et al., 2007). Moreover, since the CNCs were aligned at ± 45° angles to the tension testing clamps, the tensile yield stresses showed a decrease from the cast scaffolds due to the same “scissor” effect (Snell, 1978; Pereira and de Morais, 2004; Botelho et al., 2007). It should be noted that previous studies have shown CNC and material alignment through polarized Raman spectroscopy, when shear force is applied through either rheology, extrusion, or 3D printing (Mendez et al., 2011; Reising et al., 2012; Hausmann et al., 2018; Fallon et al., 2019). And although polarized Raman spectroscopy was not performed in this study to show CNC alignment, based on current literature references of CNC alignment properties through shear, it can be inferred that some alignment within the structure in the direction of the printing nozzle occurred, leading to the observed mechanical properties and trends (Mendez et al., 2011; Reising et al., 2012; Hausmann et al., 2018; Fallon et al., 2019). Since the 95/5 w/w composition had too low of a viscosity to print successfully, the properties could not be compared with the 95/5 w/w cast scaffold.

#### Gradient Material Scaffolds

**Figure 8:**
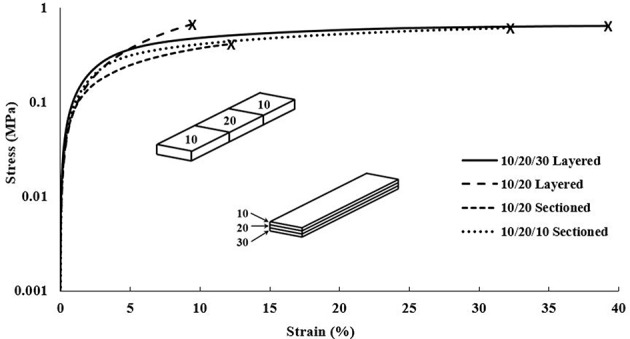
DMA stress vs. strain plot of each composition of gradient scaffolds showing strain until break. Note that the scaffolds were split into sectioned and layered designs, superimposed into the graph, with each section of the design containing a different bioink composition. The compositions used for each scaffold are shown in Table 2, for example, the 10/20/30 layered relating to 90/10, 80/20, and 70/30 w/w layers.

The DMA data for the gradient scaffolds did not show any specific trend with regards to CNC content increasing, however, demonstrated properties from all compositions used within the scaffolds, shown in Figure 8. The best mechanical properties were exhibited by the layered scaffolds, increasing the overall elastic moduli and tensile yield stresses when compared to the higher CNC compositions of the cast and single material scaffolds. The layered scaffolds show an ideal distribution of loads, in which the weaker compositions increased in mechanical toughness and the flexible compositions increased the elongation of the gradient composites (Jones, 1999; Gay, 2015; Camarero-Espinosa et al., 2016; Gao et al., 2016), shown in Table 4. The sectioned scaffolds showed a similar combination of characteristics from the use of multiple bioink compositions, however, the seams of the sections led to failure through separation (Schumacher et al., 2013). The force applied by the DMA was perpendicular to the crosslinked seams within the sectioned scaffolds, causing failure to occur at those stress points (Schumacher et al., 2013), as opposed to the layered scaffolds having equally distributed force among all of the layers (Kelly and Zweben, 2000). It should be noted that the layers did not delaminate during tensile testing. As well, the network of CNCs maintained the same alignment effects as the single material scaffolds, lending the flexibility of the “scissor” effect to the structure, while the strength of the 90/10 w/w composition was utilized (Snell, 1978; Pereira and de Morais, 2004; Botelho et al., 2007). These scaffolds demonstrated stiffness gradients that could be fine-tuned by using different bioinks of PEGDA/CNC ratios, while most other studies show the ability of porosity gradients (Woodfield et al., 2005; An et al., 2015; Bracaglia et al., 2017; Bittner et al., 2019).

Overall, the gradient scaffolds showed increased mechanical properties when compared to the cast and single material scaffolds of similar compositions. For example, the 10/20/30 layered gradient scaffold showed lower mechanical properties than the cast and single material 90/10 w/w compositions, however, they showed drastic improvements for both the 80/20 and 70/30 w/w compositions in all categories (elastic modulus, tensile yield stress, and strain at break). These results prove the ability to print multiple materials in a single scaffold, allowing for the tunability of the gradient scaffolds as a whole.

## Conclusions

Bioprinting has advanced through many challenges and limitations in the past few decades, with research focusing on improving bioprinter technology, biomaterials, cell sources and viability, vascularization, innervation, maturation, and complex 4D functionalization. Recent advances such as novel bioinks, gradient scaffolds, and stem cell differentiation within scaffolds have paved the way for complex 4D tissue scaffolds with improved reliability and functionality for modern biomedical applications. This study focused on expanding the bioprinting field through fabrication of PEGDA/CNC bioinks for extrusion bioprinting of single material and gradient scaffolds. The bioinks and scaffolds were thermally and mechanically characterized by TGA, rheology, and DMA. The final compositions of bioinks were determined, and a general trend of increasing water content and decreasing rheological yield stress with increasing CNC content was established. The 90/10, 80/20, 70/30, and 60/40 w/w bioinks showed high enough viscosities to print successful scaffolds, while the 95/5 w/w bioink not only demonstrated a low viscosity, but also showed slippage during rheology resulting in poor data. Unlike most CNC composites shown in literature (Kumar et al., 2014; Sapkota et al., 2016; Smyth et al., 2017; Frost and Foster, 2019), as the CNC content increased, the scaffolds demonstrated a decrease in both elastic modulus and yield stress, while no specific trend was observed for the strain at break. The cast scaffolds showed higher mechanical properties for the 90/10 and 80/20 w/w compositions, and lower mechanical properties for the 70/30 and 60/40 w/w compositions when compared to the single material scaffolds. The gradient scaffolds showed unique mechanical properties, utilizing the benefits of each composition to increase mechanical properties of the scaffold as a whole. The bioinks and gradient scaffolds successfully demonstrated tunability of their mechanical properties by varying CNC content within the bioink composition and the compositions used in the gradient scaffolds. This work makes strides to overcome the main disadvantages of SLA printing which consist of the inability to print multiple cell types and materials in resin form, the lack of controlled directionality of materials, and the inability to place fillers, cells, and other biological components in specific locations among the scaffolds (Fairbanks et al., 2009; Jaramillo et al., 2012; Raman and Bashir, 2015; Bishop et al., 2017; Palaganas et al., 2017; Jiang T. et al., 2019; Jiang Z. et al., 2019; Tang et al., 2019). The PEGDA/CNC bioinks and scaffolds produced in this study seek to progress biomaterials and bioprinting technologies, by transitioning SLA-dominated PEGDA bioprinting to extrusion bioprinting, in order to produce more complex, functional scaffolds for tissue engineering. Extrusion bioprinting will allow for controlled directionality, cell placement, and increased complexity of materials and cell types, improving the reliability and functionality of the scaffolds for tissue engineering applications.

## Data Availability Statement

The datasets generated for this study are available on request to the corresponding author.

## Author Contributions

BF and EF conceived of the presented idea. BF carried out bioink preparation, bioprinting of the scaffolds, thermal and mechanical characterization, and wrote the manuscript with input from all authors (BS, PT, MB, and EF). BS carried out the rheology experimentation, data analysis, and analytical calculations. PT supplied the equipment necessary to carry out the research, and contributed to the implementation of bioprinting. MB reviewed and provided feedback and edits on the written manuscript. EF supervised the project and provided critical feedback on the written manuscript. All authors discussed and proofread the manuscript.

### Conflict of Interest

PT was employed by company CELLINK^®^ LLC. The remaining authors declare that the research was conducted in the absence of any commercial or financial relationships that could be construed as a potential conflict of interest.
